# Hsa_circ_0031608: A Potential Modulator of VSMC Phenotype in the Rupture of Intracranial Aneurysms

**DOI:** 10.3389/fnmol.2022.842865

**Published:** 2022-03-11

**Authors:** Chuanchuan Wang, Yin Luo, Haishuang Tang, Yazhou Yan, Xiaozan Chang, Rui Zhao, Qiang Li, Pengfei Yang, Bo Hong, Yi Xu, Qinghai Huang, Jianmin Liu

**Affiliations:** ^1^Neurovascular Center, Changhai Hospital, Naval Medical University, Shanghai, China; ^2^Department of Biomedical Engineering, School of Life Sciences and Technology, Huazhong University of Science and Technology, Wuhan, China; ^3^Department of Neurosurgery, Naval Medical Center of PLA, Shanghai, China; ^4^Department of Neurosurgery, 971 Hospital of PLA, Qingdao, China; ^5^Department of Neurosurgery, Henan Provincial People’s Hospital, Zhengzhou, China

**Keywords:** circular RNA, intracranial aneurysm, vascular smooth muscle cell, phenotypic modulation, rupture

## Abstract

**Background and Purpose:**

Phenotypic modulation of vascular smooth muscle cells (VSMCs) plays an important role in the development of intracranial aneurysms (IAs). Growing evidence has demonstrated that circular RNAs (circRNAs) may serve as a potential modulator of VSMC phenotype in various vascular diseases. This study aimed to assess the potential function of circRNAs in the rupture of IAs and VSMC phenotypic modulation.

**Methods:**

Using surgically dissected human ruptured (*n* = 8) and unruptured (*n* = 8) IA lesions, differentially expressed circRNAs were screened by transcriptomic sequencing and verified using qRT-PCR. Based on the screened circRNA, we predicted and screened the combined miRNA and downstream mRNAs to construct circRNA-miRNA-mRNA networks. Further *in vitro* experiments were performed to investigate the relationship between the validated circRNA and the phenotypic switching of VSMCs.

**Results:**

We found 1,373 differentially expressed genes in ruptured versus unruptured aneurysms. The top five dysregulated circRNAs were selected for qRT-PCR validation. We found hsa_circ_0031608 was both highly expressed in ruptured IAs and pro-inflammatory transformation of VSMCs. Then, a regulatory circRNA-miRNA-mRNA with one circRNA node, six miRNA nodes, and 84 mRNA nodes was constructed. GO analysis and KEGG pathway enrichment analysis were performed on mRNAs in the network. Then, a PPI network was built based on these mRNAs and five hub genes were identified (FOXO3, DICER1, CCND2, IGF1R, and TNRC6B) by the cytoHubba plugin in Cytoscape software. *In vitro*, overexpression of hsa_circ_0031608 influenced the expression of VSMC phenotypic markers validated by qPCR and Western blotting. Furthermore, hsa_circ_0031608 promoted the migration and proliferation capacity of VSMCs.

**Conclusion:**

hsa_circ_0031608 regulated the phenotypic modulation of VSMCs and played an important role in the rupture of IAs. The specific mechanism should be further studied and confirmed.

## Introduction

Intracranial aneurysm (IA) is a common cerebrovascular disease with a prevalence of 3.2% (Vlak et al., 2011). Although only a minority of aneurysms rupture, the consequences of IA rupture are devastating with extremely high morbidity and mortality (Nieuwkamp et al., 2009). The preventive treatment of unruptured aneurysms with invasive procedures carries potential risks of serious complications. Owing to such a challenging clinical dilemma, identifying appropriate non-invasive treatment strategies for IAs is of paramount importance.

Although many studies have been conducted, the mechanism behind the formation and rupture of IAs is still unclear. Previous studies have revealed that phenotypic modulation of vascular smooth muscle cells (VSMCs) induced by abnormal hemodynamics and vascular inflammation may be associated with the development of IA (Kilic et al., 2005; Penn et al., 2014). Phenotypic modulation of VSMCs refers to the transition of VSMCs from a contractile phenotype to a pro-inflammatory phenotype, which is characterized by the decreased expression of Alpha-smooth muscle actin (α-SMA) and calponin, and increased expression of matrix metallopeptidases (MMPs). However, the mechanism of the phenotypic transformation described has not been fully elucidated as yet.

Circular RNA (circRNA) is a type of non-coding RNA (ncRNA) characterized by a covalently closed loop, which is formed by direct back-splicing in a non-canonical order (Chen, 2016). circRNAs are exoribonuclease-resistant and more stable than their linear transcripts (Chen, 2020). circRNAs are not just highly enriched in the brain, but they continually increase in the brain from embryonic to adult stages to regulate functions related to neuronal plasticity (You et al., 2015; van Rossum et al., 2016; Mehta et al., 2020). Additionally, altered expression of circRNAs is thought to mediate many brain diseases, including brain tumors and neurodegenerative disorders by various mechanisms, such as angiogenesis, autophagy, apoptosis, and inflammation (Han et al., 2018; Patop and Kadener, 2018; Jiang et al., 2019; Wang et al., 2019a; Liu et al., 2020b; Li et al., 2021). There is also growing evidence demonstrating the functions of circRNAs in the cerebrovascular system (Li et al., 2019; Lu et al., 2020). However, the role of circRNA in intracranial aneurysms has not yet been fully explored. Interestingly, circRNAs have been recently identified as novel modulators in the phenotypic modulation of VSMCs among studies on the cardiovascular system (Zhang and Sun, 2021). In this study, we used high-throughput sequencing to acquire circRNA profiles in human ruptured and unruptured IA tissues. Subsequently, we performed bioinformatical analysis and *in vitro* experiments to explore the potential functions of circRNAs in IA rupture and VSMC phenotype switching.

## Materials and Methods

### Patient Selection and Tissue Collection

A human subject research protocol was approved in advance by the Ethical Committee of Changhai Hospital. All participants signed informed consent before research participation. Patients were eligible for inclusion in the study: (1) aged ≥ 18 years; (2) sporadic IA diagnosed with digital subtraction angiography or CT angiography; and (3) selected by operating neurosurgeons on a basis that removal of the residual aneurysmal wall would not affect the outcome of the treatment. Additionally, patients had to have no family history of IAs and no documented history of genetic vascular disease. Clinical and surgical information were obtained from patients’ charts by the authorized study team.

Aneurysmal domes from 18 ruptured and 18 unruptured IAs were prospectively collected from patients undergoing microsurgical clipping (Supplementary Table 1). Tissue samples were collected in RNAlater (Invitrogen by Thermo Fisher Scientific) and stored at −80°C until further processing.

### RNA Isolation

Total RNA was isolated with TRIzol reagent (Invitrogen, Carlsbad, CA, United States) and quantified using the NanoDrop ND-1000 Spectrophotometer (Thermo Fisher Scientific, Waltham, MA, United States), and only good-quality RNA with an OD260/OD280 ratio between 1.8 and 2.1 was used. RNA integrity and gDNA contamination were then assessed using electrophoresis on a denaturing agarose gel.

### circRNA Sequencing and Bioinformatics Analysis

High-throughput circRNA sequencing and subsequent bioinformatics analysis were performed by CloudSeq Biotech (Shanghai, China) as follows. The raw sequence data reported in this paper have been deposited in the Genome Sequence Archive (Chen et al., 2021a) in National Genomics Data Center (Cncb-Ngdc, 2022), China National Center for Bioinformation/Beijing Institute of Genomics, Chinese Academy of Sciences (GSA-Human: HRA001908) that are publicly accessible at https://ngdc.cncb.ac.cn/gsa-human. First, linear RNAs were removed from the total RNA in each sample after treatment with RNase-R (Epicenter, Madison, WI, United States). The RNase R treated RNA was then rRNA depleted using Ribo-Zero rRNA Removal Kits (Illumina, San Diego, CA, United States). The rRNA-depleted RNA was used to construct the RNA-seq library with TruSeq Stranded Total RNA Library Prep Kit (Illumina, San Diego, CA, United States) following the manufacturer’s instructions. The quantity and quality of the library were evaluated with Agilent 2100 Bioanalyzer (Agilent Technologies, Palo Alto, CA, United States). The RNA libraries were denatured as single-stranded DNA molecules. The cDNAs were captured on Illumina Flow Cells (Illumina, San Diego, CA, United States), amplified *in situ* as clusters, and finally sequenced with 150-bp paired reads on Illumina HiSeq 4000 sequencer (Illumina, San Diego, CA, United States) according to the manufacturer’s instructions. Quality control was performed using a Q30. After 3′ adapter-trimming and low quality reads removal with Cutadapt software (v1.9.3), high quality trimmed reads were aligned to the reference genome/transcriptome with STAR software (v2.5.1b) (Dobin et al., 2013). circRNAs were detected and annotated with DCC software (v0.4.4) using two public circRNA databases: circBase and circ2Traits (Ghosal et al., 2013; Glažar et al., 2014; Cheng et al., 2016). The junction read counts were normalized, and differentially expressed circRNA analyses were performed using the edgeR software (v3.16.5) (Robinson et al., 2010). We defined the statistical criteria for selecting aberrant-expressed circRNA using an adjusted *P*-value < 0.05 with a fold change (FC) ≥ 2.0. Hierarchical Clustering was performed to show the distinguishable circRNAs expression pattern among samples.

### Quantitative Real-Time PCR Analysis

Quantitative real-time PCR (qRT-PCR) with SYBER green analysis was used to validate circRNA expression. Of the circRNAs identified, five upregulated and five downregulated exonic circRNAs were selected for validation. The housekeeping gene glyceraldehyde-3-phosphate dehydrogenase (GAPDH) was used as an endogenous control gene for normalization. The primers were designed using the “out-facing” strategy, where the circle template was amplified (Supplementary Table 2). Total RNA was reverse-transcribed into first-strand cDNA using a PrimeScript RT Reagent Kit (Perfect Real Time; TaKaRa, Osaka, Japan). Random primers were used as the RT primers for detecting circRNA and mRNA. Quantitative PCR was performed using SYBR *Premix Ex Taq* II (Tli RNaseH Plus; TaKaRa, Osaka, Japan) on the LightCycler 96^®^ (Roche) machine. Relative circRNA expression was calculated using the 2^–ΔΔCt^ method.

### Cell Culture and Phenotypic Modulation

Human brain vascular smooth muscle cells (VSMCs) were purchased from ScienCell (Catalog NO. 1100). VSMCs were resuscitated and grown in Smooth Muscle Cell Media (ScienCell) and used for the experiment after 3–5 times of passage. Tumor Necrosis Factor Alpha (TNF-α) has been shown to induce VSMC phenotypic modulation in IA pathology (Ali et al., 2013). VSMCs were treated with 20 ng/ml TNF-α for 24h to induce VSMC phenotypic modulation. Recombinant human TNF-α protein was purchased from Abcam (Catalog NO. ab259410).

### RNase R Digestion Assay

Ribonuclease R (RNase R, Epicenter, Madison, WI, United States) were used to evaluate the stability of circular RNA. A quantity of 1 ug total RNA was digested with 3 U RNase R at 37°C for 10 min. After stopping the reaction at 70°C for 10 min, circRNA and linear mRNA were detected by qPCR. Samples without RNase R treatment were used as controls.

### Nucleoplasm Separation Assay

The nuclear/cytoplasmic RNA was isolated from VSMCs using a PARIS™ Kit (Thermo Fisher Scientific) according to the protocol and subjected to qRT-PCR analysis. After washing once with phosphate-buffered saline (PBS), fresh cultured cells were resuspended in 300-μl ice-cold cell fractionation buffer, incubated on ice for 5–10 min, and then centrifuged at 500 × g for 3 min at 4°C. Then, the cytoplasmic fraction was carefully aspirated away from the nuclear pellet. Subsequently, approximately 400 μl ice-cold cell disruption buffer and an equivalent volume of 2 × lysis/binding solution were added to the nuclear pellet. After mixing by inversion, 400 μl 100% ethanol was added to the mixture. The sample mixture was then drawn into a filter cartridge. After orderly washing, centrifugation, and filtration, RNA was eluted twice with elution solution at 95°C. Finally, the isolated nuclear/cytoplasmic RNA was stored at −80°C for later use.

### Construction circRNA-miRNA-mRNA Network and Functional Annotation

Based on the circRNA-seq and validated data, we firstly searched for circRNA-targeted miRNAs in the CircInteractome database^1^ and circBank database^2^ (Dudekula et al., 2016; Liu et al., 2019). The intersection of these two datasets was assayed according to the prediction results. Secondly, we used the miRWalk bioinformatics tool^3^ to predict the target genes of the miRNAs (Sticht et al., 2018). Represented targets include those predicted for three prediction algorithms (miRDB, miRTarBase, and TargetScan) in the miRWalk program. As the final output, we have obtained the key circRNAs, targeted miRNAs, and genes, respectively, and then we can construct the circRNA-miRNA-mRNA network, which was visualized using Cytoscape software (version 3.9.0) (Shannon et al., 2003). Functional annotation was conducted using the Metascape online tool^4^. Targeted genes were mapped to pathway terms including the GO biological process (BP), cellular component (CC), molecular function (MF), and Kyoto Encyclopedia of Genes and Genomes (KEGG) Pathway. *P*-values < 0.01 were considered to indicate that the pathways or GO biological process terms were significantly enriched. Additionally, we validated the expression level of these predicted candidates in our whole transcriptome sequencing data (unpublished).

### Protein-Protein Interaction Network Analysis and Hub Gene Prediction

The STRING (Search Tool for the Retrieval of Interacting Genes; version 11.5)^5^ online tool was employed to obtain gene interactions for those target genes. Based on this protein-protein interaction (PPI) network, the hub nodes were investigated by the Maximal Clique Centrality (MCC) algorithm in CytoHubba, a plugin in Cytoscape (Chin et al., 2014). According to the order of MCC value, the top 5 were defined as hub genes. Based on these hub genes, the preliminary circRNA-miRNA-mRNA network was reconstructed as circRNA-miRNA-hub genes network.

### Transient Transfection for Overexpression of circRNA

The full-length hsa_circ_0031608 (position: chr14:35055436-35078948; spliced length: 740 nt) was cloned into the pLC5-ciR plasmid (Guangzhou Geneseed Biotech Co., Ltd., China) by *in vitro* DNA synthesis to construct the hsa_circ_0031608 overexpression vector (ov-circ_0031608). The empty pLCD5H-ciR plasmid was used as a negative control (NC). Cells at 60-70% confluence were transfected using Lipofectamine 3000 (Invitrogen, Carlsbad, United States) according to the manufacturer’s instructions.

### Wound-Healing Assay

The wound-healing assay was used to evaluate the migration rate of cells. The transfected VSMCs were seeded in a 6-well plate. After 24 h incubation, parallel wounds with similar width were scratched with a 200 μl tip. Cells were cultured in the 5% serum medium after washing away floating cells with PBS. Cell migration was observed and photographed at 0h and 24 h. The wound healing rate was measured by the way as follows: [1-(the wound area after 24 h/the starting wound area)] × 100. Wound area was calculated using the ImageJ (version 1.8.0_201) software.

### Cell Counting Kit-8 Assay

After transient transfection for at least 48 h, VSMCs were seeded in 96-well plates at a density of 2 × 10^3^ cells per well with 200 μl of the medium. The cell proliferation assays were measured by cell counting kit-8 (CCK-8) (MedChemExpress, New Jersey, United States) at a 1:10 dilution with serum-free medium every 24 h for three days, directed by the manufacturer’s protocols. Finally, OD450 of cells was shown by GraphPad Prism to reflect the ability of cell proliferation.

### Western Blotting

After scraping of experimental cells, 10 μL/mg RIPA (Beyotime, Shanghai, China) was added to the lysate. Western blotting analysis was performed using the method described in our previous study (Yan et al., 2021). The primary antibodies against MMP2, α-SMA, and β-actin were the same as those used in our previous study (Luo et al., 2021; Yan et al., 2021). Anti-Calponin antibody (13938-1-AP; Proteintech, United States) was also used. Protein bands were quantified by densitometry using ImageJ software.

### Statistical Analysis

Statistical analyses were performed using SPSS 19.0 (SPSS, Chicago, IL, United States), GraphPad Prism 5 (GraphPad Software, CA, United States), R software version 3.2.1^6^, and Microsoft Excel (Microsoft, DC, United States). Quantitative data were expressed as mean ± SD. The student’s t-test was used to analyze the comparison between two groups. A two-sided P value less than 0.05 was considered statistically significant.

## Results

### Profiles of circRNAs From Human Intracranial Aneurysm Samples

**FIGURE 1:**
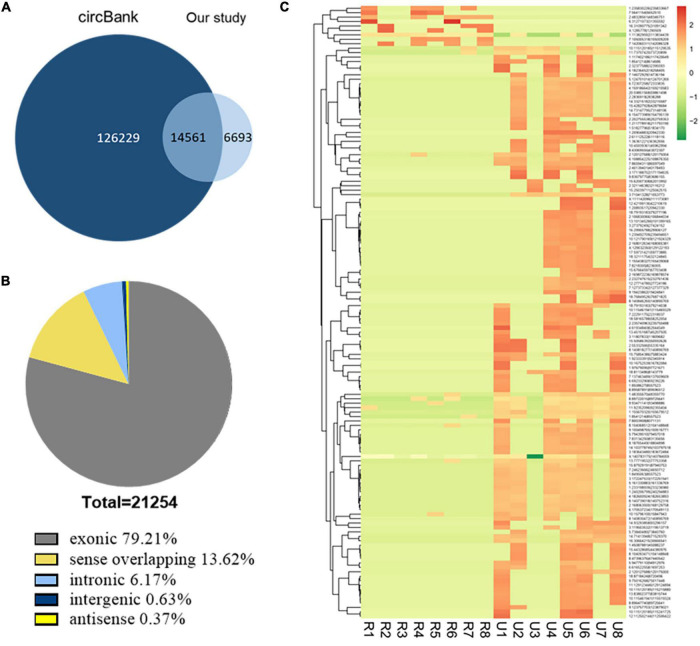
Identification of circRNAs by RNA-seq analyses in IA tissues. **(A)** Most of the circRNAs identified in our study overlapped with circBank. **(B)** Genomic origin of the circRNAs identified in IA tissues. **(C)** Clustered heat map of the differentially expressed circRNAs in eight ruptured and eight unruptured IA tissues. Rows represent circRNAs while columns represent tissues. R, ruptured; U, unruptured.

We analyzed the profiling of circRNAs in eight ruptured IAs and eight unruptured IA tissues by RNA deep sequencing (Supplementary Table 1). The sequencing statistics were described (Supplementary Table 3). A total of 21,254 circRNA transcripts were identified (Supplementary Table 4). Among them, 14,561 circRNAs (68.51%) have been identified in other studies in circBank (140,790 human circRNAs), and the other 6,693 ones (31.49%) are novel (Figure 1A). Furthermore, 16,836 circRNAs (79.21%) consisted of protein-coding exons from 6,772 host genes (Figure 1B and Supplementary Table 4), whereas smaller fractions aligned with circRNAs derived from intron lariats, intergenic circRNAs that consist of unannotated regions of the genome, antisense circRNAs transcribed from antisense regions, and sense overlapping circRNAs that originated from both exon and other sequences.

The expression analysis showed that a total of 1,373 circRNAs were differentially expressed in ruptured and unruptured IAs. The clustering heatmap illustrates the circRNA expression levels that were distinguished and clustered (Figure 1C). Among the differentially expressed circRNAs, 306 were upregulated and 1,067 downregulated (Supplementary Table 5). Among the 1,373 differentially expressed ones, 1,198 are from exons.

### Validation of the Selected circRNAs by Quantitative Real-Time PCR

**FIGURE 2:**
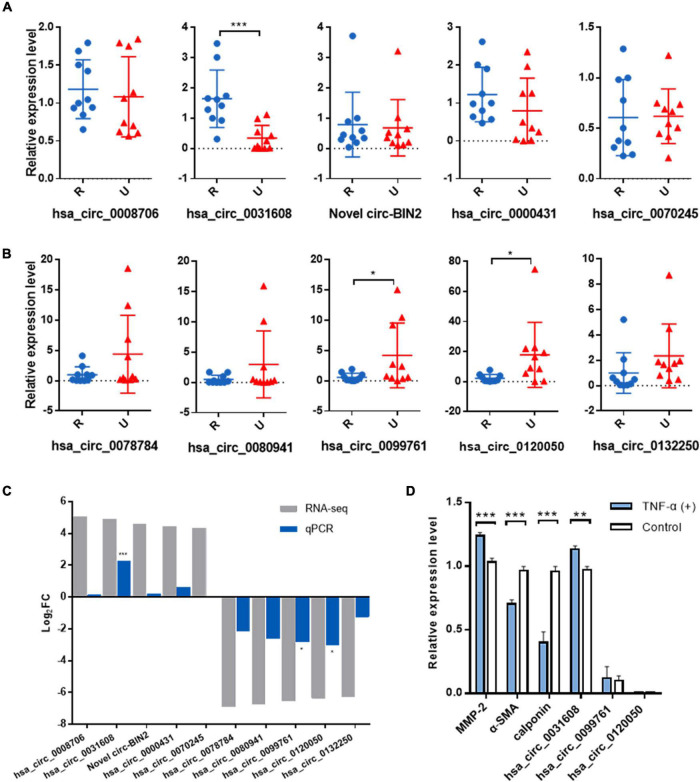
Validation of the significantly differentially expressed circRNAs in another 20 IA samples and the role of the validated circRNA in VSMC phenotypic modulation. **(A)** Relative expression of top five upregulated circRNAs by qRT-PCR. **(B)** Relative expression of top five downregulated circRNAs by qRT-PCR. **(C)** Overview of fold change in differentially expressed circRNAs in RNA-seq and qRT-PCR. **(D)** The expression for phenotype markers of VSMC and validated circRNAs were examined by qRT-PCR after TNF-α induction. *p < 0.05, **p < 0.01, and ***p < 0.001 were considered significant.

To validate the expression levels of differentially expressed circRNAs, the top five upregulated and top five downregulated exonic circRNAs were selected, and expression levels were confirmed by qRT-PCR in another 10 ruptured and 10 unruptured IAs using circRNA-specific divergent primers (Table 1 and Supplementary Tables 1, 2). The expression level of ten selected circRNAs all have similar trends with the sequencing result except hsa_circ_0070245, but only one upregulated (hsa_circ_0031608) and two downregulated circRNAs (hsa_circ_0099761 and hsa_circ_0120050) were differentially expressed significantly (*P* < 0.05; Figures 2A–C).

**TABLE 1:** A total of 10 significantly differentially expressed circRNAs identified *via* circRNA sequencing.

circRNA	Gene	logFC	*P*-value	Chr	Type
hsa_circ_0008706	PALM2-AKAP2	4.594141007	0.00101137	9	exonic
hsa_circ_0031608	SNX6	4.45325821	0.00241581	14	exonic
Novel circ-BIN2	BIN2	4.334170361	0.005128554	12	exonic
hsa_circ_0000431	DRAM1	4.207788025	0.003038399	12	exonic
hsa_circ_0070245	SEC31A	4.196592639	0.003601558	4	exonic
hsa_circ_0078784	PSMB1	−6.87973385	1.22246E-08	6	exonic
hsa_circ_0080941	PCLO	−6.728748383	4.8807E-08	7	exonic
hsa_circ_0099761	NALCN	−6.508830298	1.44645E-07	13	exonic
hsa_circ_0120050	SLC8A1	−6.275024326	9.12467E-07	2	exonic
hsa_circ_0132250	RIMS1	−6.209559626	4.00674E-08	6	exonic

### Upregulation of Hsa_circ_0031608 Was Positively Correlated With Vascular Smooth Muscle Cells Undergoing Phenotypic Modulation

We first investigated the role of the validated circRNA in VSMC phenotypic modulation. We used TNF-α to induce VSMC phenotypic modulation and analyzed the expression for phenotype markers of VSMC, (α-SMA, calponin, and MMP-2) by qRT-PCR, and our results showed that MMP-2 expression was upregulated after TNF-α induction whereas that of α-SMA and calponin was downregulated, suggesting phenotypic modulation of VSMCs. Among the validated circRNA, only hsa_circ_0031608 showed significantly increased expression after phenotypic transformation, which suggested that hsa_circ_0031608 might be involved in the phenotypic transformation process of VSMCs (Figure 2D).

### Characteristics of Hsa_circ_0031608

**FIGURE 3:**
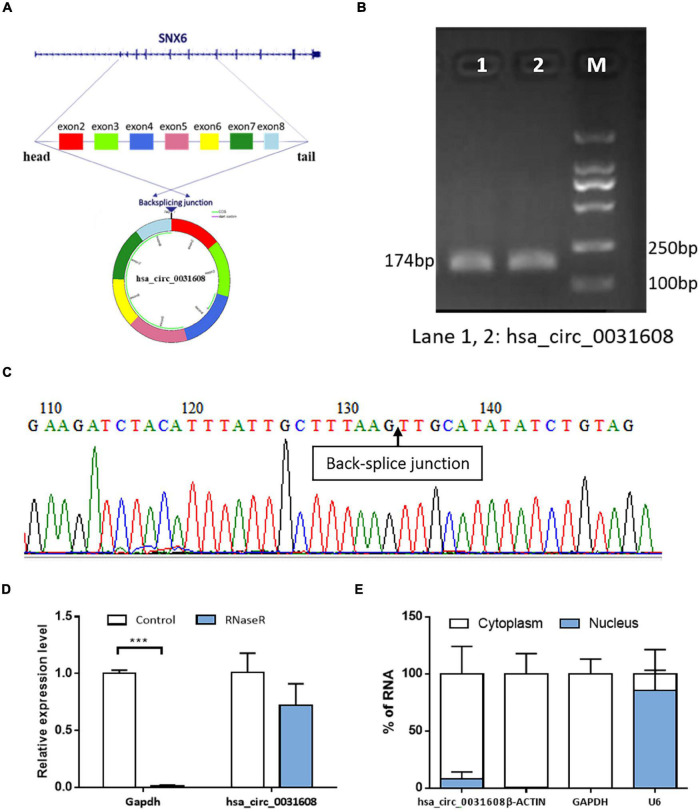
The characteristics of the circRNA hsa_circ_0031608. **(A)** Scheme illustrating the production of hsa_circ_0031608. **(B)** Verification of the size of the primer amplification product (174 bp) by agarose gel electrophoresis. **(C)** The back-splice junction of hsa_circ_0031608 was verified by Sanger sequencing using the DNA fragment from qRT-PCR. **(D)** hsa_circ_0031608 was tolerable to the degradation of RNase R. **(E)** The subcellular localization of hsa_circ_0031608 was assessed by nucleoplasm separation assay. ***p < 0.001, was considered significant.

Hsa_circ_0031608 was formed by the back splicing of exons 2-8 of the *SNX6* (NM_021249) gene located on homo sapiens chromosome 14 with a sequence length of 740 bp (Figure 3A). To verify the specificity and accuracy of the amplification procedure, the PCR amplification products were subjected to 2.5% agarose gel electrophoresis. The single electrophoresis bands were consistent with the size of the primer amplification product (Figure 3B). Sanger sequencing validated the head-to-tail junction of hsa_circ_0031608 using specific divergent primers (Figure 3C). Furthermore, RNase R was used to pretreat the RNAs, and the linear GAPDH RNA was significantly reduced. However, the expression of hsa_circ_0031608 did not decrease, which indicates that circRNA can resist digestion by RNase R due to its unique circular structure (Figure 3D). We further extracted RNA from VSMCs by nucleoplasm separation assay and found that hsa_circ_0031608 accounted for a higher proportion in the cytoplasm, suggesting that it might participate in VSMC phenotypic modulation mainly through posttranscriptional regulation (Figure 3E).

### Construction of circRNA–miRNA–mRNA Network and Functional Enrichment

**FIGURE 4:**
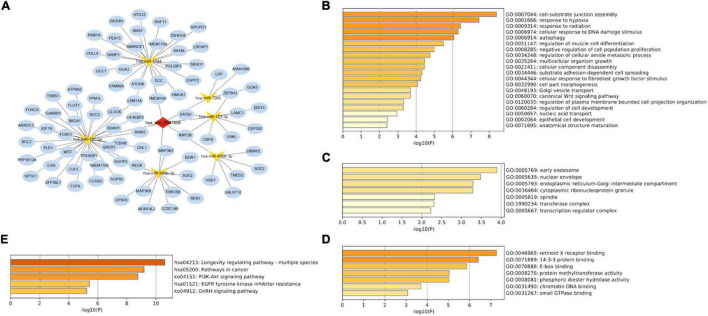
Construction of the circRNA-miRNA-mRNA regulatory network. **(A)** The network presented an initial perception of the association between the hsa_circ_0031608, the six miRNAs (hsa-miR-1184, hsa-miR-153-3p, hsa-miR-548b-3p, hsa-miR-1265, hsa-miR-450b-3p, and hsa-miR-182-5p) and 84 mRNAs. The enrichment results of these target genes were visualized by the Metascape online tool, including GO-BP **(B)**, GO-CC **(C)**, GO-MF **(D)**, and KEGG pathways **(E)**.

A total of six miRNAs were predicted by circBank and CircInteractome databases simultaneously, and the binding sites were all ≥ 3 (Table 2 and Supplementary Table 6). The target genes of these miRNAs were predicted by the miRWalk program, and 84 intersected mRNAs were selected as predicted target genes under three prediction algorithms (miRDB, miRTarBase, and TargetScan) (Supplementary Table 7). A circRNA–miRNA–mRNA network was constructed and visualized by the Cytoscape 3.8.0 software (Figure 4A).

**TABLE 2:** Hsa_circ_0031608-targeted miRNAs are predicted by circBank and CircInteractome databases.

miRNA	number of binding sites	context + score percentile
hsa-miR-1184	6	99
hsa-miR-153-3p	6/	94
hsa-miR-548b-3p	4	98
hsa-miR-1265	4	97
hsa-miR-450b-3p	4	96
hsa-miR-182-5p	3	99

To gain further insight into the potential functions of the 84 mRNAs obtained by merging the targeted mRNAs of six predicted-miRNAs, gene enrichment analysis was performed and visualized by the Metascape online tool. The GO enrichment results of BP terms were mainly enriched in cell functions, such as cellular component assembly, cellular response to hypoxia, cell differentiation, and proliferation (Figure 4B). For GO-CC enrichment terms, the most important GO terms involved in the early endosome, nuclear envelope, and endoplasmic reticulum-Golgi intermediate compartment (Figure 4C). For GO-MF terms, the majority of the enriched terms were related to protein binding, protein methyltransferase activity, and phosphoric diester hydrolase activity (Figure 4D). The enrichment of KEGG pathways demonstrated the target genes are mainly related to the pathway in cancer, the Phosphatidylinositol 3-kinase (PI3K)/Akt signaling pathway, and the epidermal growth factor receptor (EGFR) tyrosine kinase inhibitor resistance pathway (Figure 4E).

For a preliminary validation of the candidate miRNAs and genes, we checked the expression level in our whole transcriptome sequencing data (unpublished). The initial result showed that hsa-miR-153-3p is significantly downregulated among five circRNA-targeted miRNAs; three target genes (ARRDC3, FAM98A, LAMC1) are significantly upregulated (*P* < 0.05 with FC ≥ 2.0, Supplementary Table 8).

### Identification of Hub Genes in Protein-Protein Interaction Network by CytoHubba

**FIGURE 5:**
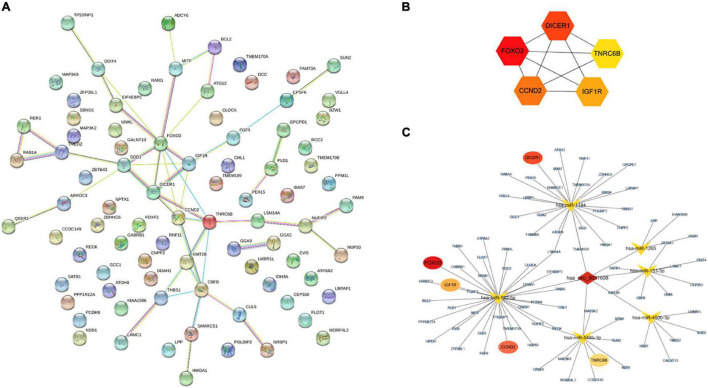
Reconstruction of the circRNA-miRNA-mRNA network. **(A)** PPI network obtained on STRING website. **(B)** Relationship network diagram of hub genes from PPI network. **(C)** The reconstructed circRNA-miRNA-hub genes network.

A PPI network based on 84 targeted genes was constructed to understand the interaction between these genes by the STRING website (Figure 5A). Then the top five hub genes (forkhead box O3 [FOXO3], DICER1, cyclin D2 [CCND2], insulin like growth factor 1 receptor [IGF1R], and trinucleotide repeat containing adaptor 6B [TNRC6B]) were calculated by the MCC method in the cytoHubba plugin (Figure 5B and Supplementary Table 9). The identified hub genes were mapped into the initial network to obtain a reconstructed network, which contains five circRNA-miRNA-mRNA regulatory axes (Figure 5C).

### Overexpression of Hsa_circ_0031608 Promoted Vascular Smooth Muscle Cell Phenotypic Modulation

**FIGURE 6:**
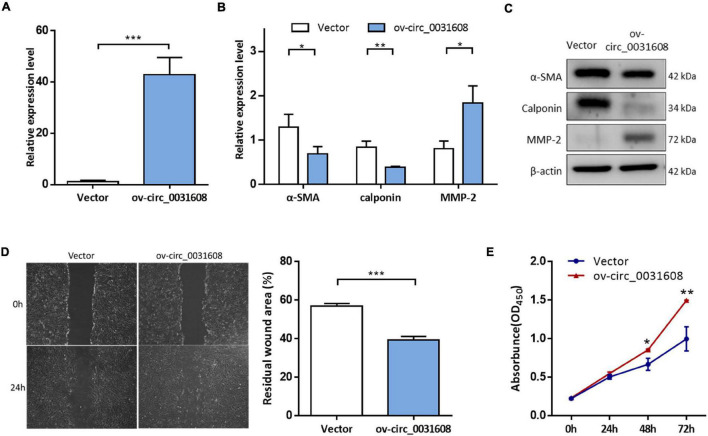
The overexpression of hsa_circ_0031608 promoted phenotypic modulation of VSMCs. **(A)** hsa_circ_0031608 abundance was examined in VSMCs transfected with vector or hsa_circ_0031608 overexpression vector (ov-circ_0031608). **(B)** mRNA expression levels of α-SMA, Calponin, and MMP-2 in VSMCs with vector or ov-circ_608 were analyzed by qRT-PCR. **(C)** Protein expression levels of α-SMA, Calponin, and MMP-2 were analyzed by Western blotting. **(D)** The migration ability of VSMCs was evaluated by wound healing assays. **(E)** Cell proliferation capacity was assessed in VSMCs *via* CCK-8 assays. *p < 0.05, **p < 0.01, and ***p < 0.001 were considered significant.

To investigate the effect of hsa_circ_0031608 on the biological behavior of VSMCs, we further explored its function by overexpressing hsa_circ_0031608 in VSMCs. Our results showed that hsa_circ_0031608 expression levels were increased in VSMCs upon transfection with ov-circ_0031608 compared to the cells transfected with the empty vector group (Figure 6A). Subsequently, we examined the expression for phenotype markers of VSMCs after hsa_circ_0031608 overexpression using qPCR and Western blotting. Our studies revealed that both α-SMA and calponin levels decreased, while MMP-2 increased upon circ_0031608 overexpression in VSMCs (Figures 6B,C). Furthermore, we examined the effect of hsa_circ_0031608 overexpression on the migration and proliferation capacity of VSMCs. The wound-healing assay revealed that the level of hsa_circ_0031608 was positively correlated with the migration ability of the cells (Figure 6D). The CCK-8 assay suggested that VSMCs upregulated in hsa_circ_0031608 promoted cell proliferation (Figure 6E). Overall, these results indicate that hsa_circ_0031608 overexpression promoted the phenotypic transformation of VSMCs.

## Discussion

In this study, we found that expression of hsa_circ_0031608 was significantly high in ruptured IAs compared to that in unruptured IAs, and also showed significantly increased expression after VSMC phenotypic transformation. The Sanger sequence analysis and RNase R digestion experiment verified that hsa_circ_0031608 is a genuine circRNA. Our subcellular localization analysis indicated that hsa_circ_0031608 resides predominantly within the cytoplasm, which suggests that circRNAs act as miRNA sponges and influence the expression of miRNA target genes. Hence, we constructed a circRNA-miRNA-mRNA network and a PPI network, aiming to screen out potential target genes involved in regulating the rupture of IAs. Additionally, we analyzed the role of hsa_circ_0031608 in VSMCs. We found that hsa_circ_0031608 overexpression enhanced the migration and proliferation capabilities of VSMCs. Meanwhile, the expression of VSMC phenotypic marker proteins was influenced after its overexpression. These results suggest that hsa_circ_0031608 plays an important role in the phenotypic transformation of VSMCs.

It is still unclear about the IA formation and rupture, but accumulating evidence underlines the important functions of circRNAs within IAs. Due to the stability of circRNA, circRNA-based biomarker discovery studies within peripheral blood were conducted (Teng et al., 2017; Huang et al., 2021). circRNA expression profile analyses of blood samples were also conducted to investigate the mechanism in the pathogenesis of IAs (Cao et al., 2021; Ma et al., 2021). Besides, tissue-based circRNA microarray or sequencing was performed by comparing IA walls and superficial temporal arteries (STAs) (Huang et al., 2019; Chen et al., 2021b). However, these circRNA biomarkers have not yet been verified experimentally. And little is known about the association between circRNAs and the rupture of IAs. A tissue-based circRNA microarray, using five unruptured and five ruptured IAs, was recently reported and revealed the role of hsa_circ_0005505 in the proliferation, migration, and apoptosis of VSMC, and indicated its potential association with the rupture of IAs (Chen et al., 2021c). Similarly, our sequencing data and *in vitro* experiments revealed hsa_circ_0031608 as a potential modulator of VSMC phenotype in the rupture of IAs. Importantly, our study not only confirms the upregulated differentially expressed level of hsa_circ_0031608 with the use of a larger sample size but also validates the authenticity of hsa_circ_0031608 with a circular structure.

Given that hsa_circ_0031608 localizes to the cytoplasm, as described above, we hypothesized that miRNA sponge activity could be a possible mechanism for its functional effects. Hence, a circRNA–miRNA–mRNA regulatory network was constructed. Functional annotation and pathway analysis indicated that the target genes were involved in the PI3K-Akt signaling pathway and EGFR tyrosine kinase inhibitor resistance pathway. We previously reported that the EGFR tyrosine kinase inhibitor regulated phenotypic modulation of VSMCs and protected rats from IA initiation (Luo et al., 2021). The PI3K-Akt signaling pathway was also demonstrated to play an important role in VSMC biology and vascular remodeling in IAs (Li and Wang, 2019). Moreover, five hub genes (FOXO3, DICER1, CCND2, IGF1R, and TNRC6B) were identified by the cytoHubba plugin based on the PPI network. Among them, FOXO3 was reported to promote VSMC phenotypic switching to accelerate aortic aneurysm formation (Lu et al., 2021). DICER1 functioned as a ribonuclease and DICER-dependent miRNAs were important for VSMC development and functioned by regulating proliferation and contractile differentiation (Albinsson et al., 2010). CCND2 was regulated by lncRNA HIF1A-AS2/miR-30e-5p in promoting VSMC proliferation (Lin et al., 2021). TNRC6B was involved in RNA binding activity and played a facilitator role in tumors (Chen et al., 2020; Liu et al., 2020a). IGF1R also played a critical role in the maintenance of normal VSMC phenotype (Siddals et al., 2011). Therefore, these hub genes may present promising therapeutic targets for combating IAs.

The mechanism that circRNAs regulate the development of IAs remains to be further studied. To date, most research has concentrated on the function of circRNAs in modulating VSMC phenotype. For example, circRNA dedicator of cytokinesis 1 (circDOCK1), a circRNA first described in colorectal cancer, was detected a lower expression level in IA tissues compared with middle meningeal artery tissues by real-time PCR. Three studies revealed that circ_0020397 overexpression could regulate VSMC biological functions *via* miR-138/kinase insert domain receptor (KDR), miR-409-3p/MCL1, and miR-502-5p/gremlin 1 (GREM1) axis (Wang et al., 2019b; Ding et al., 2021; Yin and Liu, 2021). Similarly, given the underexpression in IA tissue and the peripheral blood, circRNA ADP ribosylation factor interacting protein 2 (circ-ARFIP2) was studied in human umbilical artery smooth muscle cells (HUASMCs) and overexpression of circ-ARFIP2 in HUASMCs enhanced proliferation, migration, and invasion of HUASMCs by targeting the miR-338-3p/KDR axis (Qin et al., 2021). In the present study, considering the high expression of hsa_circ_0031608 in IA tissues and VSMCs undergoing phenotypic modulation, hsa_circ_0031608 overexpression experiments were performed in VSMCs and showed a profound promotion of VSMC phenotypic modulation, implying that upregulated hsa_circ_0031608 might lead to the enhanced vascular remodeling in IAs. Regrettably, the predicted downstream miRNA and gene targets of the circRNA–miRNA–mRNA regulatory network was not examined and validated in the present study, which is the main defect and more in-depth experiments are required in the following study.

Our study has several limitations that should prompt further validation and study. Firstly, the expression of predicted miRNA and mRNA candidates still needs to be further confirmed. Second, VSMCs express a number of common markers, including but not limited to α-SMA and Calponin. For example, Myh11 is the most specific marker of the VSMC but not evaluated in this study, which may affect the accuracy of VSMC phenotype identification. Future research should measure more additional markers including Myh11, SM22α, and others. Third, this study just investigated the effect of overexpression of hsa_circ_0031608 in VSMC phenotypic switch. The knock-down experiment should be performed if the inhibition of hsa_circ_0031608 can block or reverse VSMC phenotypic modulation in the future. Lastly, this study only included *in vitro* cell experiments, and further *in vivo* studies of hsa_circ_0031608 in aneurysm rupture are still needed.

In conclusion, the results of this study imply that hsa_circ_0031608 promote the phenotypic modulation of VSMCs and play an important role in the rupture of IAs. Accordingly, hsa_circ_0031608 may serve as a potential target for IA therapy and novel drug research.

## Data Availability Statement

The datasets presented in this study can be found in online repositories. The names of the repository/repositories and accession number(s) can be found in the article/Supplementary Material.

## Ethics Statement

The studies involving human participants were reviewed and approved by Committee on Ethics of Medicine, Navy Medical University, PLA. The patients/participants provided their written informed consent to participate in this study. Written informed consent was obtained from the individual(s) for the publication of any potentially identifiable images or data included in this article.

## Author Contributions

CW, YL, HT, YY, and XC performed the experiments. RZ, QL, PY, BH, and YX were responsible for collection of samples and clinical data. QH and JL contributed to the conception or design of the work. CW drafted the work. All authors approved the final version of the manuscript.

## Conflict of Interest

The authors declare that the research was conducted in the absence of any commercial or financial relationships that could be construed as a potential conflict of interest.

## Publisher’s Note

All claims expressed in this article are solely those of the authors and do not necessarily represent those of their affiliated organizations, or those of the publisher, the editors and the reviewers. Any product that may be evaluated in this article, or claim that may be made by its manufacturer, is not guaranteed or endorsed by the publisher.

## Footnotes

1
https://circinteractome.nia.nih.gov

2
http://www.circbank.cn

3
http://mirwalk.umm.uni-heidelberg.de/

4
http://metascape.org/gp/

5
https://string-db.org/cgi/input.pl

6
http://www.r-project.org/
